# Comparative Effectiveness Randomized Clinical Trial Using Next-generation Microbial Sequencing to Direct Prophylactic Antibiotic Choice Before Urologic Stone Lithotripsy Using an Interprofessional Model

**DOI:** 10.1016/j.euros.2023.09.008

**Published:** 2023-09-28

**Authors:** Michael A. Liss, Kelly R. Reveles, Craig D. Tipton, Jonathan Gelfond, Timothy Tseng

**Affiliations:** a Department of Urology, University of Texas Health San Antonio, San Antonio, TX, USA; b College of Pharmacy, University of Texas, Austin, TX, USA; c Pharmacotherapy Education & Research Center, University of Texas Health San Antonio, San Antonio, TX, USA; d RTL Genomics, MicroGen DX, Lubbock, TX, USA; e Department of Biological Sciences, Texas Tech University, Lubbock, TX, USA; f Department of Population Sciences, University of Texas Health San Antonio, San Antonio, TX, USA

**Keywords:** Kidney stone, Lithotripsy, Next-generation sequencing, Microbiome, Infection

## Abstract

**Background:**

Next-generation sequencing (NGS) methods for microbial profiling have increased sensitivity to detect urinary pathogens.

**Objective:**

To determine whether NGS microbial profiling can be used to guide antibiotic prophylaxis and reduce infection compared with the standard of care.

**Design, setting, and participants:**

A prospective randomized controlled clinical trial of patients undergoing urologic stone interventions at an academic health center from December 2019 to January 2022 was conducted. Urine was collected at the preoperative visit for standard culture and intervention NGS diagnostics. Evaluable patients were culture negative, met 2-wk follow-up, and did not cancel surgery. Of 240 individuals (control = 121, intervention = 119), 83 control and 74 intervention patients were evaluable.

**Intervention:**

Microbial findings (paired quantitative polymerase chain reaction and NGS) were sent to an infectious disease pharmacist to recommend prophylactic antimicrobial regimen.

**Outcome measurements and statistical analysis:**

The primary outcome was postoperative urinary infection within the follow-up period (Fisher’s exact test). The primary outcome was analyzed by modified intent-to-treat (mITT) and per-protocol analyses. Secondary endpoints considered included positive culture concordance, antibiotic use, and adverse events. Additional post hoc analyses investigated factors contributing to infection (univariate logistic regression).

**Results and limitations:**

The intervention significantly reduced postsurgical urinary infection risk by 7.1% (–0.73%, 15%) compared with the standard of care in the mITT analysis (1.4% vs 8.4%, *p* = 0.045) or by 8.5% (0.88%, 16%) compared with the per-protocol analysis (0% vs 8.5%, *p* = 0.032). NGS-guided treatment altered the distribution of antibiotics used (*p* = 0.025), and antibiotics poorly matched with NGS findings were associated with increased infection odds (odds ratio [OR] = 5.9, *p* = 0.046). Women were at greater odds to develop infection (OR = 10, *p* = 0.03) and possessed differentiated microbial profiles (*p* < 0.001).

**Conclusions:**

Urinary microbial NGS-guided antibiotic prophylaxis before endoscopic urologic stone lithotripsy improves antibiotic selection to reduce healthcare-associated urinary infections; however, treatment efficacy may be limited by the ability to adhere to the recommended protocol.

**Patient summary:**

We investigated whether microbial DNA sequencing could improve the selection of antibiotics before kidney stone surgery in patients not known to have any bacteria in the urine on standard culture. We found that using microbe DNA to guide antibiotic choices decreased postoperative infection rate and may encourage individualized use of available antibiotics.

## Take Home Message

We investigated whether microbial DNA sequencing could improve the selection of antibiotics before kidney stone surgery in patients not known to have any bacteria in the urine on standard culture. We found that the use of microbe DNA to guide antibiotic choices decreased postoperative infection rate and may encourage individualized use of available antibiotics.

## 1 Introduction

Globally, there were 1394 incident cases of kidney stones per 100 000 population in 2019 [1]. Cole et al [2] noted that one in 40 patients is hospitalized with an infection-related complication following ureteroscopy for urologic stones. The risk of infectious complications after urologic stone endoscopic surgery is approximately 7%, but can range from 2% to 20% [3], [4], [5], [6], [7], [8], [9]. Nearly one in seven patients present with an unplanned postprocedural visit after urologic stone surgery, largely infection related [10].

However, the infection rates stated previously occur despite antibiotic prophylaxis using preoperative cultures [11]. There remains considerable variation among surgeons regarding antibiotic prophylaxis choices and can be a major issue leading to unplanned hospital admissions for infection [12], [13], [14]. It is known that preoperative urine cultures have low sensitivity for predicting infectious complications of urologic stone procedures [15], [16], [17], [18]. Next-generation sequencing (NGS) methods have been used extensively in research to describe the human microbiome and are beginning to guide antibiotic choices in other specialties (eg, orthopedic implant-associated infections) [19], [20], [21]. Therefore, we hypothesized that we could change the antibiotic prophylaxis standard of care by using microbial DNA and associated resistance profiles to select antibiotics.

Herein, we use a randomized controlled clinical trial to determine the feasibility and utility of microbial NGS-guided antibiotic prophylaxis in culture-negative patients, which would reduce postoperative infection and maintain acceptable antibiotic stewardship.

## 2 Patients and methods

### 2.1 Trial design

We designed a randomized controlled single-center trial in San Antonio, TX, USA. The trial protocol and statistical analysis plan were designed by the trial investigators. In the control group, the physician was blinded to the results. We chose not to blind the surgeon in the intervention arm to maintain safety for the patient in allowing the surgeon to ultimately decide the best antibiotic for surgery and NGS that would augment clinical support information. The trial was sponsored by the MicroGen DX. The study was approved by the institutional review board (IRB) at the University of Texas Health San Antonio (HSC20190678H) and registered at clinicaltrials.gov (NCT04404855). Trial oversight was provided by the principal investigator (M.A.L.) and statistician (J.G.) along with the local data safety and monitoring committee with a biannual review.

### 2.2 Participants

We recruited patients at the time of their preoperative appointment, before planned urologic stone removal procedure from December 18, 2019 to January 28, 2022. The eligibility criteria included patients planning to undergo kidney or bladder stone removal surgery using endoscopy including ureteroscopy and percutaneous nephrolithotomy or any other transurethral procedure, aged 18 yr or older, and able to give informed consent. We excluded patients if they were unwilling to provide informed consent and were aged <18 yr, and those with a positive urine culture. Patients with ureteral stents were included.

### 2.3 Treatment allocation

We performed randomization using a random number generator to achieve simple 1:1 allocation. Once generated, we created individual envelopes, and study staff pulled an envelope after the participant signed the consent. We did not blind the research staff to the randomization groups. However, the treating surgeon in the control arm was blinded to the NGS result. The treating physicians used their preferred standard of care in the control group as per the American Urological Association white paper and were given 1 h before surgery [22], [23]. All patients with ureteral or renal pelvic stones underwent ureteral stenting at the time of surgery. We chose to randomize patients immediately due to improved logistics to groups knowing that cancelations and positive urine cultures may be prominent.

### 2.4 Interventions

#### 2.4.1 Urine collection and processing

Approximately 30–50 ml of whole urine was collected for a standard of care urine culture corresponding to the patients’ preoperative visit. We obtained approximately 5–10 ml of leftover urine for research and shipped overnight to MicroGen DX (Lubbock, TX, USA), a CAP-accredited and CLIA-licensed clinical diagnostic laboratory, for analysis. Deidentified samples were sent to MicroGen DX in accordance with their guidelines. In the event the urine culture was performed >45 d before the urologic procedure, the treating physician asked the patient to provide an additional urine sample. The commercial paired NGS and quantitative polymerase chain reaction (qPCR) panel (UROKEY; MicroGen DX) consisted of a multispecies qPCR panel, multitarget antibiotic resistance gene (ARG) panel, (bacterial) V1-V2 16S rRNA gene sequencing, and (fungal) ITS1-2 sequencing assays, as reported previously [24], [25]. Subsequent analyses are based on the microbiological findings delivered in real time to the interdisciplinary care team. Taxonomic assignments are reported to the species level where possible, but due to recognized greater uncertainty at species-level classification, species epithets should be interpreted with caution [26]. See the Supplementary material for details on laboratory processes and bioinformatics.

#### 2.4.2 Urine result interpretation

We considered a urine culture to be positive if there was identification of an organism with or without a resultant antibiotic resistance profile and irrespective of symptoms. The patient would then be given an antibiotic to treat the culture-identified organism and would be excluded. If the culture was reported as no growth, normal flora, or possible contamination and had a low urine white blood cell count (<10 wbc/hpf) that was not sent for speciation, the culture was considered normal and would proceed to NGS-directed prophylaxis. In the control arm, we collected NGS samples before randomization and processed for secondary outcomes.

#### 2.4.3 Antibiotic selection

Our interprofessional model includes the use of urinary NGS interpreted by both an infectious disease pharmacist and a urologist to guide antibiotic selection before surgery. We performed a pilot study on 20 patients before the clinical trial design and noted that NGS identifies a targetable bacterium in up to 50% of negative urine cultures prior to urologic stone surgery [27]. However, when we presented NGS data to urologists, depending on the NGS report, 25–63% of urologists broadened antibiotic coverage leading to substandard antibiotic stewardship. The pilot study influenced us to include a pharmacist to assist with reading NGS results and providing suggestions to maintain antibiotic stewardship. Therefore, NGS results and patient baseline characteristics (including concomitant medications and allergies) for the intervention group are sent to the infectious disease pharmacist (K.R.) to provide an antibiotic recommendation. Antibiotic prophylaxis was chosen based on the assessment of documented patient allergies, and organisms and antimicrobial resistance genes identified using polymerase chain reaction (PCR) and NGS. We prioritized organisms that are known common urologic pathogens (eg, *Escherichia coli*, *Klebsiella* species, *Proteus* species, *Enterococcus* species, and *Pseudomonas* species) to target for antimicrobial coverage, especially those with a high bacterial load on PCR or the highest percentage contribution to the microbial community on NGS. We did not target other organisms identified as likely commensals or contaminants (predominantly anaerobes) for antibiotic coverage. We preferentially selected antimicrobials that achieve adequate urinary concentrations (eg, beta-lactams, fluoroquinolones, trimethoprim-sulfamethoxazole, and aminoglycosides). Of these, we chose the agent that had the minimum spectrum of activity to target common urologic pathogens identified. If a resistance gene was detected to the selected antimicrobial, we chose the next antimicrobial with the minimum spectrum of activity against identified organisms. If no organisms or resistance genes were identified, we selected cefazolin as the prophylactic antimicrobial of choice. The antibiotic recommendation and the NGS results were then provided to the treating physician. The physician utilized these tools to select the best antibiotic for the case but was not required to abide by the pharmacist's suggestion. In the control group, physicians selected the prophylactic antimicrobial regimen based on the standard of care as per the American Urological Association white paper [22].

### 2.5 Outcome measures

Our primary endpoint was a clinically determined urinary tract infection (UTI) determined by a provider and treated with an antibiotic within 14 d after surgery. The pragmatic endpoint was chosen due to the challenges in collecting urine culture for infection in individuals located in other cities or hospital systems. The endpoint was attained via phone calls to all patients approximately 14 d after surgery, along with a clinical chart review. We documented if the patient had secondary contact with a medical provider via phone call, office visit, or emergency room (ER) visit to determine whether any additional antibiotics were prescribed. Any patient with diagnosis codes representing clinical infection of UTI, pyelonephritis, or sepsis thought to be of a urinary source was documented as having an infection. The secondary outcomes included the total number of microbes identified on NGS, culture results, positive culture organisms, antibiotic stewardship (number of antibiotics used), and patient-reported adverse events (any symptoms that patient related to the antibiotic—rash, nausea, or vomiting).

### 2.6 Sample size calculation

The primary hypothesis was that infection rates within 14 d of surgery would be 11% in the control arm and 1% in the intervention arm, based on our preliminary data. We calculated a sample size of 63 individuals per group (ie, a total sample size of 126) to achieve 80% power to detect a 10% difference in infection rate using a one-sided test with an alpha = 0.05. Owing to coronavirus disease (COVID) issues and higher than expected dropout rate, an IRB amendment was obtained to increase enrollment, and with a 30% dropout rate, our new target enrollment was 240 individuals to obtain the targeted number of patients.

### 2.7 Statistical analysis

Our primary outcome was infection within 14 d after surgery and was compared between groups using the one-sided Fisher’s exact test, based on the hypothesis that NGS-guided antibiotic prophylaxis would be superior to the standard of care and would reduce the rate of infection. A brief descriptive analysis was presented to compare concordance between microbes detected in excluded culture-positive samples between the two diagnostic modalities. Integrating NGS into treatment decision-making has been argued to risk increasing antibiotic usage and/or to increase efficacy by better identifying pathogens, thus personalizing antibiotic choice. Multiple antibiotic stewardship secondary endpoints intended to quantify how integrating NGS changes antibiotic usage from the standard of care, including use of any prophylactic antibiotic, reported side-effect incidence, prophylaxis augmentation (more than one antibiotic used), escalation (apparent broad-spectrum increase, here defined as any vancomycin or piperacillin-tazobactam usage), ER visits resulting in additional antibiotic use, and any combination of augmentation, escalation, and ER visit with additional antibiotics, all of which were treated as binary outcomes and analyzed by two-sided Fisher’s exact test. Lastly, regarding stewardship, the unique antibiotics used between arms were quantified and analyzed for nonrandom distribution by the chi-square test. Baseline demographics were compared between the intervention and standard of care groups using the chi-square test, Fisher’s exact test, Student *t* test, or Wilcoxon rank sum test, as appropriate. Post hoc and microbiome analyses are described in the Supplementary material.

## 3 Results

### 3.1 Enrollment and participant characteristics

**Fig. 1:**
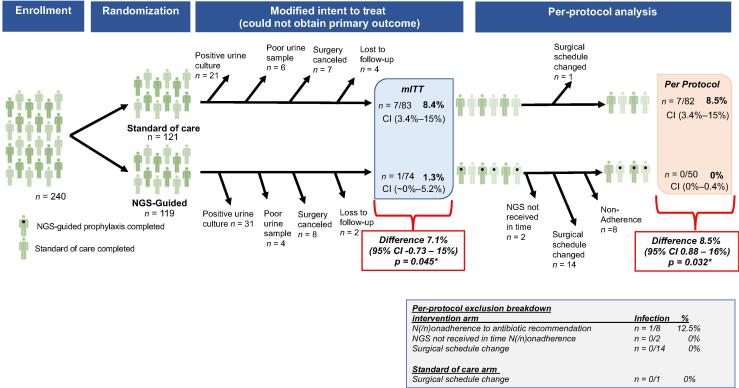
Graphic consort diagram of randomized clinical trial and modified intent-to-treat analysis. We enrolled 240 patients from a single institution in a phase 2 randomized trial. Patients were randomized at the time of urine collection in the clinic at the time of surgery scheduling. Only those patients who had a negative urine culture were randomized, leading to many cases of exclusion upfront. We report a significant reduction in infections in a modified intent-to-treat analysis using a one-tailed Fischer’s exact test. The differences between groups were compared using a two-sample test for equality of proportions. CI = confidence interval; mITT = modified intent to treat; NGS = next-generation sequencing.

We enrolled 240 individuals after IRB-approved consent process and performed the procedure at a median of 13 d from enrollment urine collection. After randomization, the intervention arm included 119 individuals and the control arm included 121 individuals. Nonevaluable patients were excluded for the following reasons: positive cultures (*n* = 52), no/poor urine provided for culture (*n* = 10), canceled surgery (*n* = 15), or loss to follow-up (*n* = 6; Fig. 1). Following nonevaluable exclusions, the intervention arm included 74 and the control arm included 83 individuals. Evaluable patient baseline characteristics included in the analysis are displayed in Table 1, and additional deviations from the protocol are marked in Figure 1. The participants were well matched in terms of baseline demographics, comorbidities, and surgical factors.

**Table 1:** Baseline characteristics of evaluable patients

Characteristic	Control (*N* = 83) a	Intervention (*N* = 74) a
Age	51 (40, 64)	52 (40, 62)
Gender (female), *n* (%)	38 (46)	29 (39)
BMI	32 (27, 37)	32 (28, 37)
Unknown	2	2
Race, *n* (%)		
White	77 (93)	63 (85)
Asian	2 (2.4)	2 (2.7)
Black or African American	0 (0)	6 (8.1)
Unknown or other	4 (4.8)	3 (4.1)
Hispanic	43 (52)	28 (38)
Surgery type, *n* (%)		
URS	67 (81)	63 (85)
Other	3 (3.6)	4 (5.4)
PCNL	13 (16)	7 (9.5)
Diabetes	19 (23)	25 (34)
Stent prior to surgery, *n* (%)	18 (22)	14 (19)
Catheter prior to surgery, *n* (%)	3 (3.6)	1 (1.4)
UTI at presentation with stone, *n* (%)	22 (27)	24 (32)
Number of current medications	5 (3, 7)	5 (3, 9)
Unknown	9	7

BMI = body mass index; IQR = interquartile range; PCNL = percutaneous nephrolithotomy; URS = ureteroscopy; UTI = urinary tract infection.

a Median (IQR); *n* (%).

### 3.2 Primary outcome

Postoperative infection was the primary endpoint. Several departures from the protocol were observed during the trial, which disproportionately impacted the intervention arm (graphic consort diagram; Fig. 1). To investigate the effectiveness of incorporating NGS into antibiotic treatment decision, a modified intent-to-treat (mITT) analysis was performed, which included all evaluable patients regardless of adherence to the protocol. As per the mITT analysis, postoperative infection decreased significantly by 7.1% (–0.73%, 15%) in the intervention compared with the control arm (*p* = 0.045; Fig. 1). In the intervention arm, the single patient who developed a postoperative infection was one of the eight patients who was nonadherent and not given the NGS-recommended antibiotic (see Supplementary Fig. 1 for a more detailed view of the preoperative findings in the eight patients with postoperative infection). When repeated per protocol with perfect adherence to NGS, we noted zero infections and an 8.5% (0.88%, 16%) absolute decrease in the intervention arm (*p* = 0.032).

### 3.3 Culture results and postoperative infections

Of the patients, 22% (52/240; intervention 31, control 21) had positive urine cultures and were excluded. For those with a positive culture and complete follow-up, 10.7% (three of 28) and 11.1% (two of 18) in the intervention and control arms, respectively, had postoperative infections. Of the patients with a positive culture and follow-up data, 13% (six of 46) had fungi detectable on NGS and all were *Candida* (*Candida glabrata*, four patients; *Candida albicans*, three; and *Candida tropicalis*, two), one of which would develop an infection. NGS microbial profiling identified a median of three species per sample compared with one species per culture. Additionally, NGS identified matching organisms from the initial urine culture in the preoperative period in 88% (37/42) of comparable specimens. Of the five cultures in which NGS did not detect the positive culture organism, the specific cultured bacteria were *Pseudomonas aeruginosa*, *Enterobacter cloacea*, *Klebsiella oxytoca*, *Staphylococcus aureus*, and *Streptococcus* Viridans group.

### 3.4 Antibiotic stewardship

**Fig. 2:**
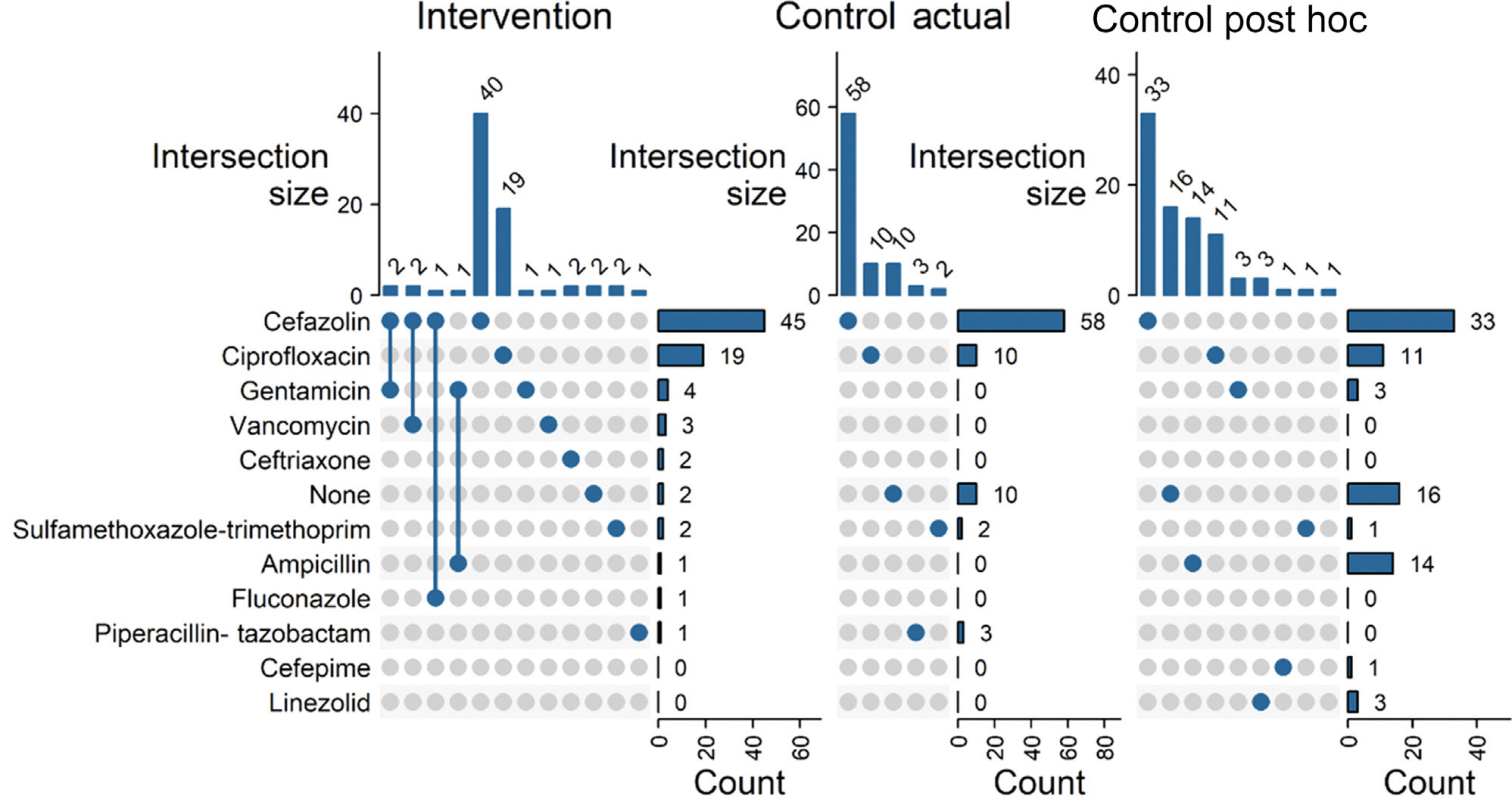
UpSet plots comparing antibiotic use. The UpSet plots show differential pattern of antibiotic usage between the intervention (left), control as used (middle), and control post hoc antibiotic recommendation (right) comparison sets. Points connected by lines indicate where combination antibiotics were used during treatment. Intersection bar plots show the number of times a specific antibiotic was used alone or in combination with another, whereas the right-hand bar plots show the number of times an antibiotic was used in total.

Patients receiving prophylaxis received the standard <24-h dose. In the NGS intervention arm, use of any prophylactic antibiotic increased (absolute) 9% from control and use of multiple prophylactic antibiotics increased 8.1% (*p* < 0.05; Table 2), while no significant change was found in terms of escalation, antibiotic side-effect incidence, or use of additional antibiotics for treating postoperative infection. The overall increased rate of any antibiotic being used for prophylaxis does not explain the difference in infection rate between arms as every patient who developed a postoperative infection was given an antibiotic (Supplementary Fig. 1). The use of additional antibiotics within 2-wk follow-up was marginally higher in the control group by 5.8%, although nonsignificant (*p* = 0.12). Additional antibiotics included ciprofloxacin (four), third-generation cephalosporins (three), nitrofurantoin (one), and Bactrim (one). When considering any augmentation, escalation, or ER visit–related stewardship event, two arms were nearly identical (Table 2). Antibiotic usage patterns differed significantly between the intervention and control arms (chi-square; *p* = 0.025; Fig. 2). Qualitatively, a decrease in the use of the primary empiric antibiotic cefazolin was observed in the intervention arm compared with the control arm. The intervention arm used eight unique antibiotics compared to four in the control arm, indicating an increased variety of antibiotic usage (Fig. 2).

**Table 2:** Antibiotic stewardship endpoints compared for each arm

Stewardship Endpoint	Control^a^	Intervention^a^	Difference^b^	95% CI^b^,^c^	P-Value^d^
Any prophylaxis ABX, *n* (%)	73 (88)	72 (97)	–9.3	–19, –0.15	0.035 *
ABX-reported side effect, *n* (%)	1 (1.2)	0 (0)	1.2	–2.3, 4.8	>0.9
Prophylaxis augmented (>1 ABX), *n* (%)	0 (0)	6 (8.1)	–8.1	–16, –0.61	0.010 *
Prophylaxis escalated (apparent broad spectrum), *n* (%)	3 (3.6)	4 (5.4)	–1.8	–9.6, 6.0	0.7
ER visit with additional ABX, *n* (%)	6 (7.2)	1 (1.4)	5.9	–1.6, 13	0.12
Any augmentation, escalation, or ER visit, *n* (%)	9 (11)	9 (12)	–1.3	–13, 10	0.8

ABX = antibiotic; ER = emergency room.

a *n* (%).

b Two sample test for equality of proportions.

c CI = confidence interval.

d Fisher's exact test.

### 3.5 Adverse events

No patients had antibiotic adverse reactions in the intervention arm. Only one patient who was allocated to the control group had a reaction to the antibiotics in which hypoglycemia attributed to a cefazolin dose of 3 g. She is a 49-yr-old female of Hispanic ethnicity with body mass index >50. The NGS result showed *C. albicans* and presented with a clinical infection, and no final cultures were reported.

### 3.6 Post hoc analysis

**Fig. 3:**
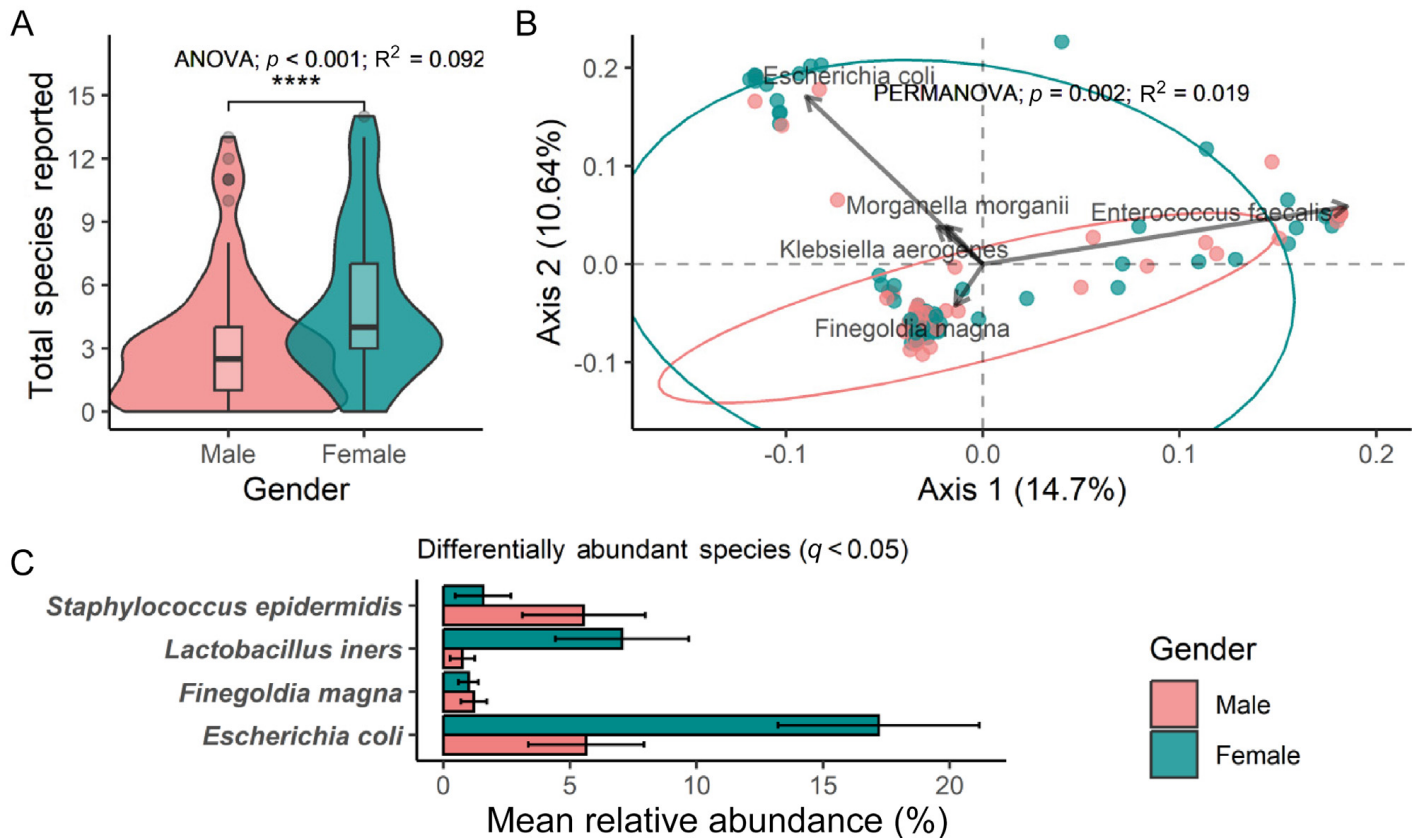
Differential microbial profiles by gender at baseline. (A) The total number of species quantified by both qPCR and NGS shown as per patient-reported gender. (B) For patients with NGS-positive 16S rRNA results, bacterial relative abundances were summarized by Bray-Curtis dissimilarity and clustered naively by PCoA; 95% confidence ellipses were added to show group-wise clustering of male versus female baseline measurements. A species-level biplot was added to show the top five species maximally correlated to axes 1 and 2. The arrow length is proportional to correlational strength. (C) Following ANCOM, four species were found to be significantly different in abundance between genders, after multiple test correction (*q* < 0.05). Error bars indicate standard error around each mean relative abundance. ANOVA = analysis of variance; NGS = next-generation sequencing; PCoA = principal coordinate analysis; qPCR = quantitative polymerase chain reaction.

Detailed results are provided in the Supplementary material. In summary, we performed exploratory post hoc analyses to consider factors that may have contributed to the observed infection rates. Women were found to have 10× higher odds of postoperative infection than men (*p* = 0.03; Table 3). Where antibiotics were inconsistent with NGS-guided infectious disease recommendations, patients had 5.9× higher odds of developing a postoperative infection (10.9% vs 2.2%, *p* = 0.04; Table 3). We noted ARG presence in 38% (59/157) and multidrug resistance (2+ ARGs) in 59% (35/59) of these, but similar distributions between the randomization arms (Supplementary Table 1). Overall, 85% (133/157) patients were found to have additional microbiological findings by paired qPCR and NGS, with a mean of 3.9 species being identified per sample (Supplementary Fig. 2). Lastly, women were found to have a significantly differentiated urinary microbial profile from men (Fig. 3).

**Table 3:** Post hoc analysis of factors associated with infection

Characteristic	Infection summary		Univariate logistic regression			
	0 (*N* = 149) a	1 (*N* = 8) a	*N*	OR	95% CI	*p* value
Poor ABX coverage, *n* (%)	50 (34)	6 (75)	157	5.94	1.31, 41.6	0.033 *
Age	51 (40, 63)	54 (52, 61)	157	1.02	0.97, 1.07	0.4
Gender (female)	60 (40)	7 (88)	157	10.4	1.78, 197	0.031 *
BMI	32 (27, 37)	30 (28, 37)	153	1.04	0.96, 1.12	0.3
Race			157			
White	133 (89)	7 (88)		–	–	
Asian	4 (2.7)	0 (0)		0.00		>0.9
Black or African American	5 (3.4)	1 (12)		3.80	0.19, 28.3	0.3
Unknown or Other	7 (4.7)	0 (0)		0.00		>0.9
Hispanic	66 (44)	5 (62)	157	2.10	0.50, 10.5	0.3
Surgery type			157			
URS	123 (83)	7 (88)		–	–	
Other	7 (4.7)	0 (0)		0.00		>0.9
PCNL	19 (13)	1 (12)		0.92	0.05, 5.62	>0.9
Diabetes	43 (29)	1 (12)	157	0.35	0.02, 2.06	0.3
Stent prior to surgery	30 (20)	2 (25)	157	1.32	0.19, 6.08	0.7
Catheter prior to surgery	4 (2.7)	0 (0)	157	0.00		>0.9
UTI at presentation with stone	42 (28)	4 (50)	157	2.55	0.58, 11.2	0.2
Number of medications	5 (3, 8)	4 (2, 8)	141	0.97	0.75, 1.22	0.8

ABX = antibiotic; BMI = body mass index; CI = confidence interval; IQR = interquartile range; OR = odds ratio; PCNL = percutaneous nephrolithotomy; URS = ureteroscopy; UTI = urinary tract infection.

a *n* (%); median (IQR).

## 4 Discussion

We report the first clinical trial utilizing NGS microbial identification to guide antimicrobial prophylaxis before urologic stone surgery to reduce postoperative infections. Our study shows feasibility and utility as significant clinical improvements by presenting a practical alternative to empiric therapies to prevent postsurgical infections. The results of a systematic review suggest that preoperative urine cultures have a poor predictive value and accuracy for infective organism in the renal stone [28]. We also anticipate that infection rates may increase over time; therefore, NGS may provide a solution to these issues using an individualized approach.

We hypothesized that microbes causative of a postoperative infection may be present at low numbers detected by DNA rather than culture before surgery; thus, tailoring prophylactic antibiotics to the preoperative urinary microbes may reduce the risk of infection. Stracy et al [29] performed whole genome sequencing of 1113 pre- and post-treatment infectious isolates for a retrospective analysis where they reported that many patients who failed treatment (ie, recurrent infection) were found to already possess microbial resistance to the antibiotics given, suggesting that molecular diagnostics improve antibiotic selection and reduce treatment complications.

**Fig. 4:**
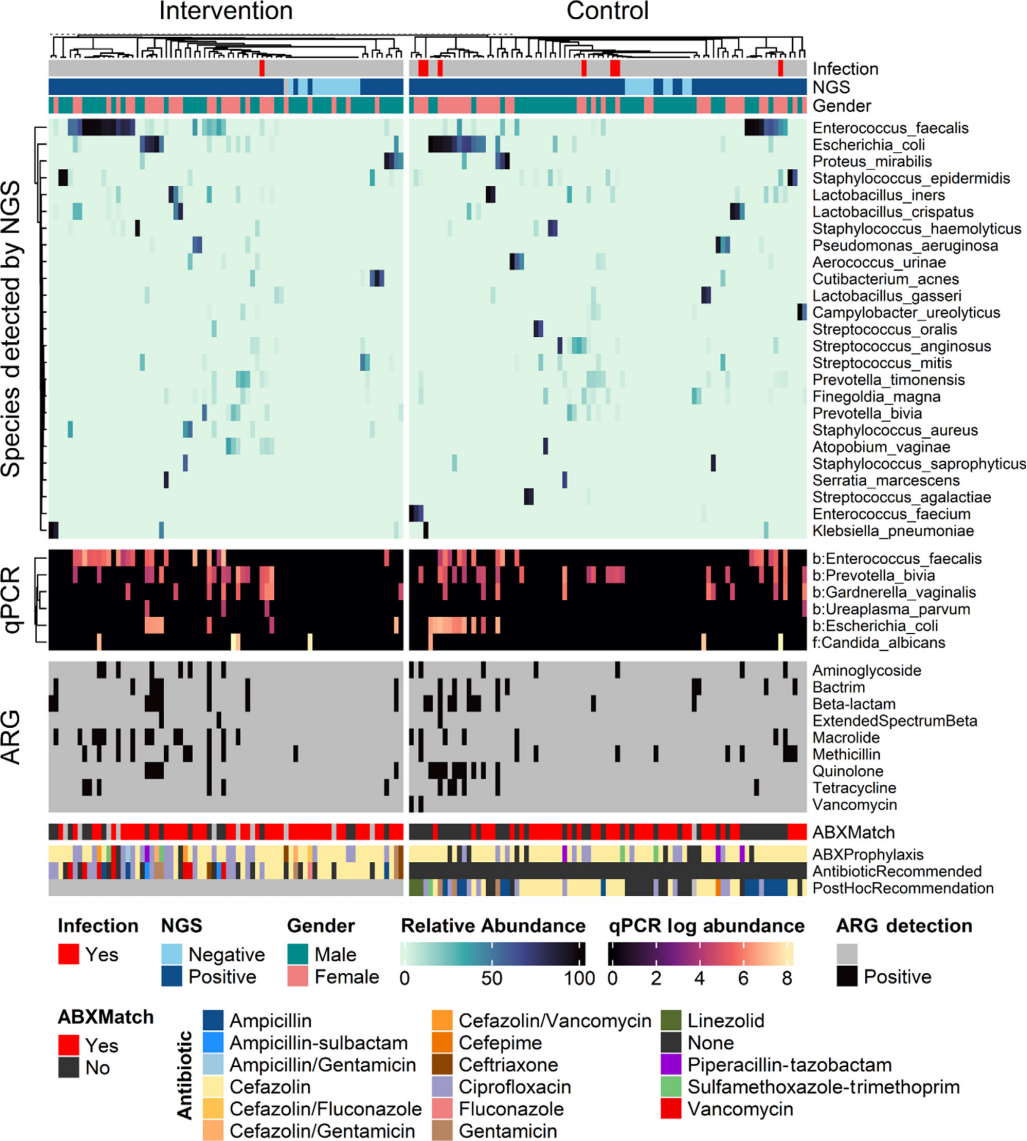
Heatmap of trial results. Heatmap shows findings per paired 16S rRNA sequencing and qPCR panel used to guide antibiotic recommendations in culture-negative patients undergoing urologic stone intervention procedures. Infection relates to occurrence of postoperative infections within the follow-up period, documented by visit to emergency room. NGS (positive or negative) relates to the findings by the commercial NGS provider MicroGen DX. Negative samples had no findings by sequencing or qPCR. NGS species are presented as reported in reports to physician and by relative abundance; here, the top 25 bacteria out of 153 total are shown. The qPCR species are shown as log-transformed abundance, with top six of 16 observed here. A prefix of b: versus f: is used to indicate bacterial versus fungal detections. Following are the findings of qPCR assays that report the detection of common antibiotic resistance–associated genes by the class of antibiotic with detected resistance. Antibiotic prophylaxis (ABXProphylaxis) shows the actual antibiotic(s) given per patient, which may differ from recommendations. The AntibioticRecommended row shows antibiotics recommended for the intervention arm by an ID pharmacist. The PostHocRecommendation row shows recommendations for a treating antibiotic made after the study had concluded, to determine how often treatment may have been changed in the control arm. Lastly, the ABXMatch column shows where the actual antibiotic(s) given in the study was consistent with the recommendation column. ABX = antibiotic; ARG = antibiotic resistance gene; ID = infectious disease; NGS = next-generation sequencing; qPCR = quantitative polymerase chain reaction.

Here, microbial NGS was used to target antibiotic prophylaxis using a wider selection of antibiotics (Fig. 4) compared with the standard of care, which relied more heavily on empiric recommendations. Concerning antibiotic stewardship, NGS-guided therapy was augmented to use more than one antibiotic (8.1%) or escalated to apparent broad-spectrum antibiotics (5.4%) in similar proportions to the number of control group patients who were prescribed additional antibiotics upon visiting the ER (7.2%). The usage of any antibiotic was greater in the NGS intervention arm (97% vs 88%); however, the different usage rate does not account for the difference in the postoperative infection rate as all eight patients who developed infection were given an antibiotic (primarily cefazolin; Supplementary Fig. 1). Six of the eight (75%) patients were given an antibiotic inconsistent with the NGS findings. For antibiotic stewardship and microbial NGS, the apparent tradeoff involved increasing antibiotic use, however reducing the rate of postoperative infection and, potentially, the rate at which patients are given additional rounds of antibiotics to combat preventable infection.

We showed that women are much more likely to have postoperative clinical infection symptoms and have more species identified despite a negative urine culture than men. Patients with a positive preoperative urine culture had a postoperative infection rate of 10.8% (five of 46) despite culture-directed antibiotics, which was slightly higher than but not statistically different from the control group infection rate (8.4%, seven of 83).

Interpretation must be mindful of limitations in the design and implementation of the current study. Beginning with trial design, we excluded patients with a positive culture who would instead receive the standard of care treatment. However, these patients were randomized and excluded later, which led to our definition of an “evaluable” population that can introduce a potential for bias. In future studies, we suggest inclusion of culture-positive patients with stratification in the protocol. Next, protocol nonadherence was a major issue exacerbated by the COVID pandemic, which disproportionately impacted the NGS-guided treatment arm because the results in this arm were time sensitive, whereas the control group was blinded to the results, so surgery rescheduling was less impactful. Protocol deviations included surgical cancellations, rescheduling, delays in NGS molecular findings being received, study coordinator staffing and scheduling issues, and miscommunication between study staff and treating physicians (eg, treating physician using empiric therapy instead of the recommended prophylaxis). Despite the noted issues, our mITT analysis found a significantly reduced postoperative infection rate and was coupled with the differential use of available antibiotics, suggesting that NGS microbial diagnostics can be applied in a practical setting to improve patient care. Lastly, we chose a clinical definition of infection as our primary outcome rather than culture-confirmed UTI. We considered any interaction with a medical provider that led to a prescription of further antibiotics an adequate and clinically meaningful endpoint. Culture confirmation could be also biased due to the variation in follow-up for patients with a suspected infection (eg, teleconference visit during COVID, outside emergency care, urgent care not linked to our system, antibiotics provided prior to culture collection, etc.). While we did not perform a formal cost:benefit analysis in this study, the commercial MicroGen DX assay used here was available for $200 per test at the time of the study, which included overnight priority shipping within the USA, laboratory processing, and reporting of results to providers within 3–5 d (average). A septic event increases the cost of ureteroscopy by $31 843 in one analysis of over 100 000 patients undergoing ureteroscopy at an incidence rate of 5% [30].

## 5 Conclusions

In conclusion, using NGS-guided antibiotic prophylaxis in those with a negative culture before urologic stone lithotripsy shows promise to prevent postsurgical infections. Using an interprofessional model with an infectious disease pharmacist, we were able to attain antimicrobial stewardship by minimizing the use of additional antibiotics or broadening the antibiotic spectrum. The data suggest proceeding with larger multi-institutional trials with more inclusive enrollment criteria.

  ***Author contributions*:** Michael A. Liss had full access to all the data in the study and takes responsibility for the integrity of the data and the accuracy of the data analysis.

  *Study concept and design*: Liss, Reveles, Tseng.

*Acquisition of data*: Liss, Reveles, Tseng.

*Analysis and interpretation of data*: Liss, Reveles.

*Drafting of the manuscript*: Liss.

*Critical revision of the manuscript for important intellectual content*: Liss, Reveles, Tseng.

*Statistical analysis*: Liss, Tipton, Gelfond.

*Obtaining funding*: Liss.

*Administrative, technical, or material support*: Liss, Tseng.

*Supervision*: Liss, Reveles, Gelfond, Tseng.

*Other*: None.

  ***Financial disclosures:*** Michael A. Liss certifies that all conflicts of interest, including specific financial interests and relationships and affiliations relevant to the subject matter or materials discussed in the manuscript (eg, employment/affiliation, grants or funding, consultancies, honoraria, stock ownership or options, expert testimony, royalties, or patents filed, received, or pending), are the following: Michael A. Liss, Kelly R. Reveles, and Timothy Tseng were recipients of funding to conduct this investigator initiated clinical trial. Other than grant support funding for the trial presented in this manuscript, these authors have no other conflicts of interest with the company in the form of other funding, financial support, nonfinancial support, or investment. Craig D. Tipton works for an affiliate of the sponsor MicroGen DX and was needed for his expertise in microbiome bioinformatics.

  ***Funding/Support and role of the sponsor*:** The research is an investigator-initiated trial supported by MicroGen DX through funding the trial and providing the NGS reports. Michael A. Liss conceived, designed, and conducted the trial without assistance from MicroGen DX. RTL, a subsidiary of MicroGen DX, had access to the data to perform the microbial bioinformatics analysis (Craig D. Tipton). An independent statistician (Jonathan Gelfond) performed statistical analysis.
