# Supplementary material for: Comparative Effectiveness Randomized Clinical Trial Using Next-generation Microbial Sequencing to Direct Prophylactic Antibiotic Choice Before Urologic Stone Lithotripsy Using an Interprofessional Model
Source: Eur Urol Open Sci. 2023 Sep 28;57:74–83. doi: 10.1016/j.euros.2023.09.008 (PMC10658407; doi:10.1016/j.euros.2023.09.008)
Supplement: Supplementary data 1 [file mmc1.docx]

**Supplementary material**

**METHODS**

**Laboratory Processes**

Urine specimens were collected via midstream catch and shipped overnight (without cold packing) to the MicroGenDX laboratory (Lubbock, TX, USA). MicroGenDX provides a comprehensive service for quantitative PCR and NGS microbial taxonomic profiling of clinical specimens. After receiving, samples are immediately prepped for DNA extraction using the Zymo MagBead 96 DNA/RNA kit (Zymo Research, Tustin, CA, USA). Samples were mechanically lysed using Zirconium oxide beads (0.5mm) and the Qiagen TissueLyser. The lysate was extracted for total DNA following the Zymo MagBead 96 DNA/RNA kit’s protocol on the KingFisher FLEX (ThermoFisher, Grand Island, NY, USA). PCR amplification was selective for the 16S rRNA hypervariable regions V1-V2 using primers 28F (GAGTTTGATCNTGGCTCAG) and 388R (GCTGCCTCCCGTAGGAGT), or for fungal targeting ITS3-4 using ITS3F (GCATCGATGAAGAACGCAGC) and ITS4R (GCATCGATGAAGAACGCAGC) primers. PCR reactions were conducted on ABI Veriti thermocyclers (Applied Biosystems, Carlsbad, CA, USA) with a thermal profile consisting of 5-minute denaturation step at 95˚C, 35 cycles of 94˚C for 30 seconds, 52˚C for 40 seconds, and 72˚C for 60 seconds, and a final extension step of 72˚C for 10 minutes. PCR products were combined based on qualitative band strength to form the pooled amplicon libraries and size selection was performed using Agencourt AMPure XP beads (Beckman Coulter, Indianapolis, Indiana, USA) and Qiagen Minelute Kits (Qiagen). Pooled libraries were quantified using a Qubit 3.0 fluorometer (Thermo Fisher Scientific, Waltham, MA, USA). Paired end sequencing (2x250) was conducted on an Illumina MiSeq (Illumina, San Diego, CA, USA).

A proprietary quantitative PCR (qPCR) panel using Roche Lightcycler 480 platform was used to estimate total bacterial load and test for presence of common ARGs, listed here: aminoglycosides [ant-la / aph3], potentiated sulfonamides [Sul I/Sul II], beta lactams [SHV / TEM], extended spectrum beta lactam [CTX-M], carbapenem [NDM / KPC / OXA], macrolides [ermB], methicillin [mecA], quinolones [qnr / gyrA], tetracycline [tetB / tetM], and vancomycin [vanA]).

**Bioinformatic Processing, Quality Control, and Reporting**

Sequence data were processed using pipelines maintained by MicroGenDX for bioinformatic processing of sequence data, summarization, and reporting to physicians. Denoising of sequence reads, chimera detection, and paired read assembly were conducted using Usearch7 [1], UCHIME [2], and PEAR [3], respectively. Quality filtered and assembled reads were clustered into operational taxonomic units (OTUs) at 97% sequence similarity threshold using the UPARSE algorithm [4]. OTU assignment then used an in-house curated taxonomic reference database. Additional Contaminant screening and quality filtering is performed by proprietary process before sending summary pdf reports to healthcare providers. Briefly, contaminant screening includes comparison of samples to negative extraction and no template controls. Reporting imposes a 2% relative abundance filter, where taxa must be more abundant than 2% within a sample to be reported by amplicon sequencing. Raw data (fastq) have been uploaded to the NCBI Sequence Read Archive (SRA) under bioproject PRJNA907321 and additional data necessary to reproduce analyses presented within the manuscript can be found in Supplementary File 1.

**ANALYSIS**

***Post Hoc Analysis:*** Exploratory post-hoc analyses further considered factors which may have contributed to the observed infection rate. First, univariate logistic regression was used to screen all baseline characteristics independently to determine if any available patient factors (significant associations shown in **Table 3**), with significant factors then being included in a multivariate model. Women were found to have 10X higher odds of post operative infection compared to men (p = 0.03, **Table 3**), accounting for 7 (88%) of infections despite composing only 43% of the evaluable patient population. No other demographic variables were significantly associated with post operative infection by independent logistic regression.

Control arm patients were still profiled by NGS, though the molecular profiling was not used to guide or change treatment. After completion of the study, the ID pharmacist was blinded to infection outcome and tasked with reviewing control patient profiles to recommend a single antibiotic. Across both arms, the ID recommendations were then compared against the antibiotics given and scored on whether the antibiotic given matched with the recommendation. For simplicity, the pharmacist had an option to score “Cefazolin or None” to indicate that recommendation would not have changed the empiric determination. The guiding hypothesis of this post-hoc analysis was that infection incidence would be greater where antibiotics were poorly matched based on NGS molecular profiling. Indeed, 6 of 55 (10.9%) overall cases where antibiotics given were inconsistent with NGS-guided ID recommendation developed a post operative infection compared to 2 of 90 (2.2%) who received antibiotic treatment matching an NGS-guided recommendation (p = 0.046, **Table 3**). Related, women (50.0%) were 57.7% more likely than men (31.7%) to not receive an antibiotic therapy consistent with recommendations based on NGS microbial profiles (p = 0.037). Gender and antibiotic matching with NGS recommendations were confounded in the current study, whereas when included in simultaneously in a multivariable regression model only gender remained statistically significant (p < 0.05, Table 3).

Considering antimicrobial resistance patterns, antibiotic resistance was predicted by detection of at least one known ARG in 38% (59/157) of the evaluable patients and multi-drug resistance (2+ ARGs) was predicted in 59% (35/59) of these individuals. ARG detection and number of ARGs per patient was mostly balanced between each arm (**Supplementary** **Table 1**), with the exception that macrolide resistance was quantified slightly higher in the intervention arm (p = 0.054). The three most common antibiotic resistances study-wide were to beta-lactams, macrolides, and methicillin. Notably, genes quantified as part of the test for beta-lactam resistance (TEM, SHV), are known to confer greater tolerance to 1st generation cephalosporins (e.g., cefazolin) which was the primary antibiotic class used as empiric therapy in the present study [5,6].

***Comparison of observed microbiota*:** Overall, 85% (133/157) patients were found to have additional microbiological findings by paired qPCR and NGS. The top 25 quantified bacteria per NGS (16s rRNA sequencing) and top 6 by qPCR panel are shown in **Figure 1**. Including NGS negative samples, a mean 3.9 species were identified per sample (**Supplementary** **Fig. 2**). Here, one goal was to investigate whether species were differentially distributed across relevant study factors, including randomization arm and gender, considering that women were more likely to develop a post operative infection. Considering NGS positivity (i.e., positive detection of at least one organism), no difference was observed in rates between each arm of study (p = 0.37). There was no difference in the number of species detected between randomization arms (p = 0.72) nor in overall microbial taxonomy when assessing variation in Bray-Curtis distances (p = 0.23, R2 < 0.01). Next, bacteria found in at least 10% of samples were screened for differential abundance by ANCOM. *E. faecalis* and *F. magna* were found to be non-randomly distributed across arms (2 of 7 screened, **Supplementary Fig. 3A**), more abundant in the intervention and control, respectively. None of these bacteria were significantly associated with development of post op infection, however some species qualitatively were found associated with post op infection at a higher rate (**Supplementary Fig. 3B**). These findings indicate that microbial profiles were generally well-matched on initial screening between the two randomization arms.

Comparing genders, women were found to have a 15-point higher rate of NGS positivity (94%) compared to men (79%, p = 0.01). Similarly, significantly more cumulative species were observed per sample from women compared to men (**Fig. 4A**). A PERMANOVA was performed to investigate the relative importance of study factors to variation in observed microbiota per patient and to evaluate whether there were group-wise differences in the randomized arms which could alternatively explain the greater infection rate in the control cohort [7]. All previous factors listed in **Table 1** were evaluated in a multivariable PERMANOVA model, whereas only gender was associated to significant change in the baseline observable microbiota (p = 0.002, R2 = 0.019, **Fig. 4B**). ANCOM procedure was used to screen bacterial species between gender, where three species were found to be differentially abundant (q < 0.05, **Fig. 4C**). Of these, Escherichia coli and Lactobacillus iners were more common among women, compared to Staphylococcus epidermidis and Finegoldia magna which were more abundant in men.

**References**

[1] Edgar RC. Search and clustering orders of magnitude faster than BLAST. Bioinformatics. 2010;26:2460-1.

[2] Edgar RC, Haas BJ, Clemente JC, Quince C, Knight R. UCHIME improves sensitivity and speed of chimera detection. Bioinformatics. 2011;27:2194-200.

[3] Zhang J, Kobert K, Flouri T, Stamatakis A. PEAR: a fast and accurate Illumina Paired-End reAd mergeR. Bioinformatics. 2014;30:614-20.

[4] Edgar RC. UPARSE: highly accurate OTU sequences from microbial amplicon reads. Nat Methods. 2013;10:996-8.

[5] Palzkill T. Structural and Mechanistic Basis for Extended-Spectrum Drug-Resistance Mutations in Altering the Specificity of TEM, CTX-M, and KPC β-lactamases. Frontiers in Molecular Biosciences. 2018;5.

[6] Liakopoulos A, van den Bunt G, Geurts Y, Bootsma MCJ, Toleman M, Ceccarelli D, et al. High Prevalence of Intra-Familial Co-colonization by Extended-Spectrum Cephalosporin Resistant Enterobacteriaceae in Preschool Children and Their Parents in Dutch Households. Front Microbiol. 2018;9:293.

[7] Anderson MJ. Permutational Multivariate Analysis of Variance (PERMANOVA). Wiley StatsRef: Statistics Reference Online. p. 1-15.

**Supplementary Tables and Figures**

**Supplementary Table 1.** Distribution and Count of Predicted Antimicrobial Resistance

| **Detected Resistance** | **Overall**, N = 157^1^ | **Control**, N = 83^1^ | **Intervention**, N = 74^1^ |
| --- | --- | --- | --- |
| Aminoglycoside | 15 (9.6%) | 7 (8.4%) | 8 (11%) |
| Bactrim | 17 (11%) | 9 (11%) | 8 (11%) |
| Beta-lactam | 20 (13%) | 11 (13%) | 9 (12%) |
| ExtendedSpectrumBeta | 3 (1.9%) | 1 (1.2%) | 2 (2.7%) |
| Macrolide | 21 (13%) | 7 (8.4%) | 14 (19%) |
| Methicillin | 20 (13%) | 10 (12%) | 10 (14%) |
| Quinolone | 16 (10%) | 10 (12%) | 6 (8.1%) |
| Tetracycline | 12 (7.6%) | 6 (7.2%) | 6 (8.1%) |
| Vancomycin | 2 (1.3%) | 2 (2.4%) | 0 (0%) |
| DrugResistance |  |  |  |
| 0 | 98 (62%) | 51 (61%) | 47 (64%) |
| 1 | 24 (15%) | 13 (16%) | 11 (15%) |
| 2 | 17 (11%) | 12 (14%) | 5 (6.8%) |
| 3 | 9 (5.7%) | 3 (3.6%) | 6 (8.1%) |
| 4 | 6 (3.8%) | 3 (3.6%) | 3 (4.1%) |
| 5 | 2 (1.3%) | 1 (1.2%) | 1 (1.4%) |
| 7 | 1 (0.6%) | 0 (0%) | 1 (1.4%) |
| ^1^n (%) | | | |

**Supplementary Figure 1**. Post operative infection summary.


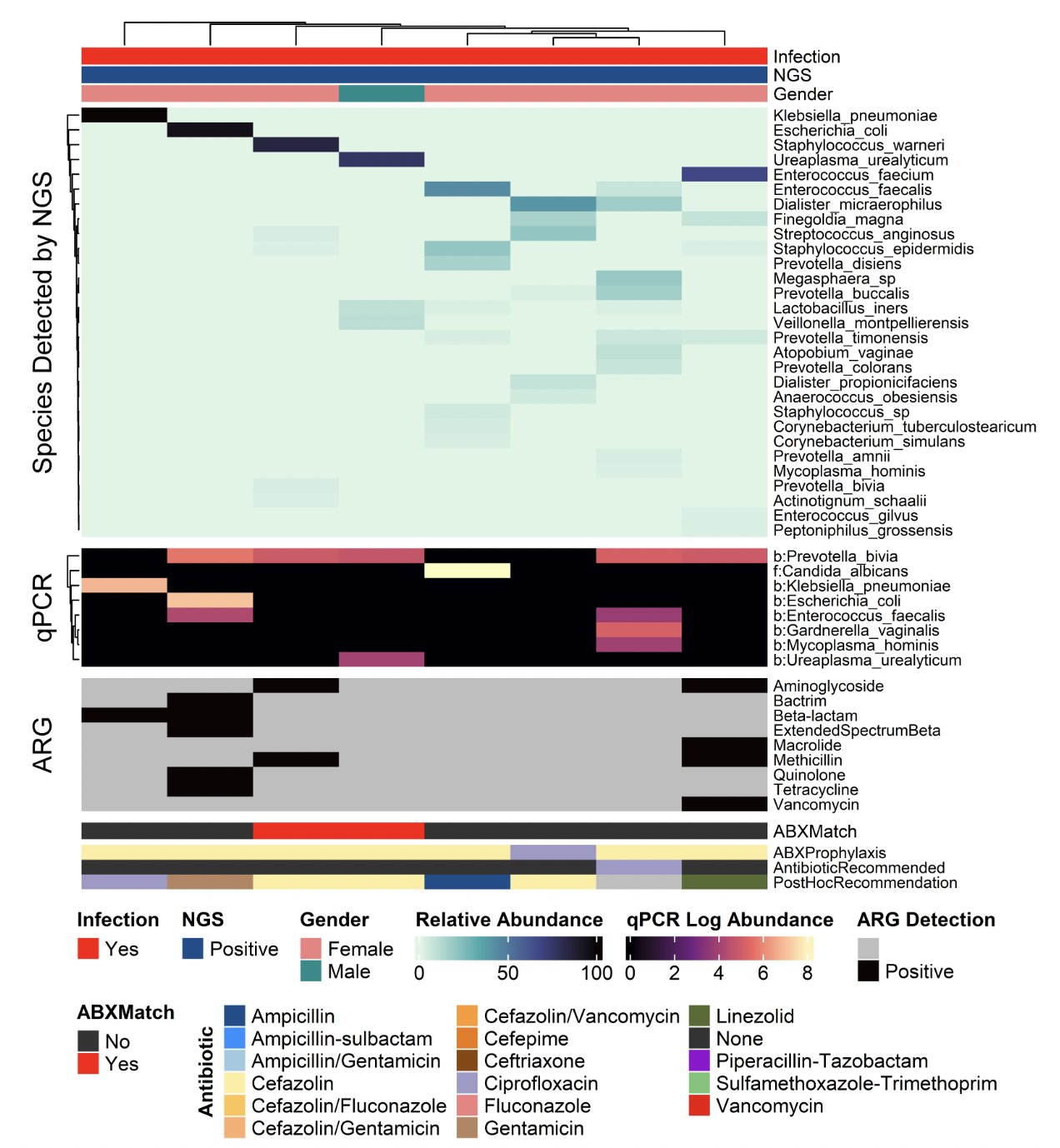


**Supplementary Figure 1.** Subset heatmap of Figure 1, focused on individuals who developed a post operative infection, regardless of arm. Here, all species are shown where there was at least one detection in the infected group.

**Supplementary Figure 2.** Number of microbial species detected.


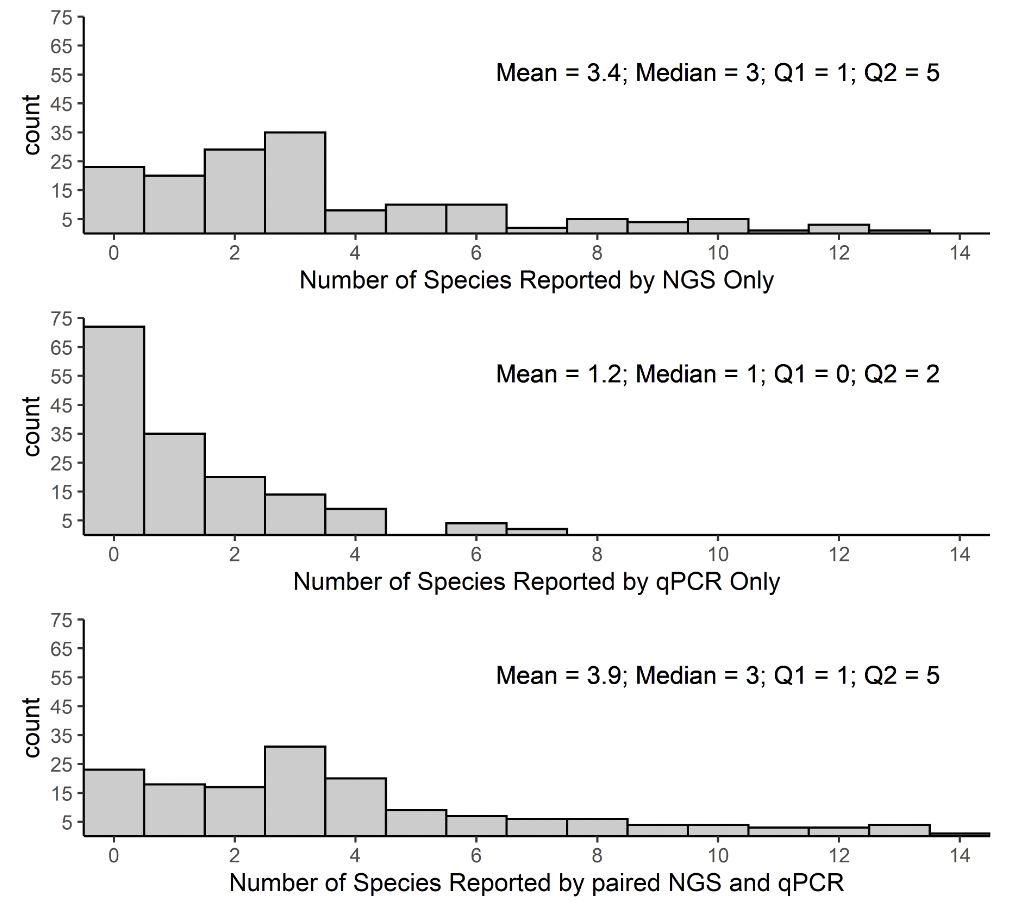
 **Supplementary Figure 2.** Distributions of number of microbial species detected by NGS alone, qPCR alone, and both.

**Supplementary Figure 3**. Next Generation Sequencing most common bacteria detected.


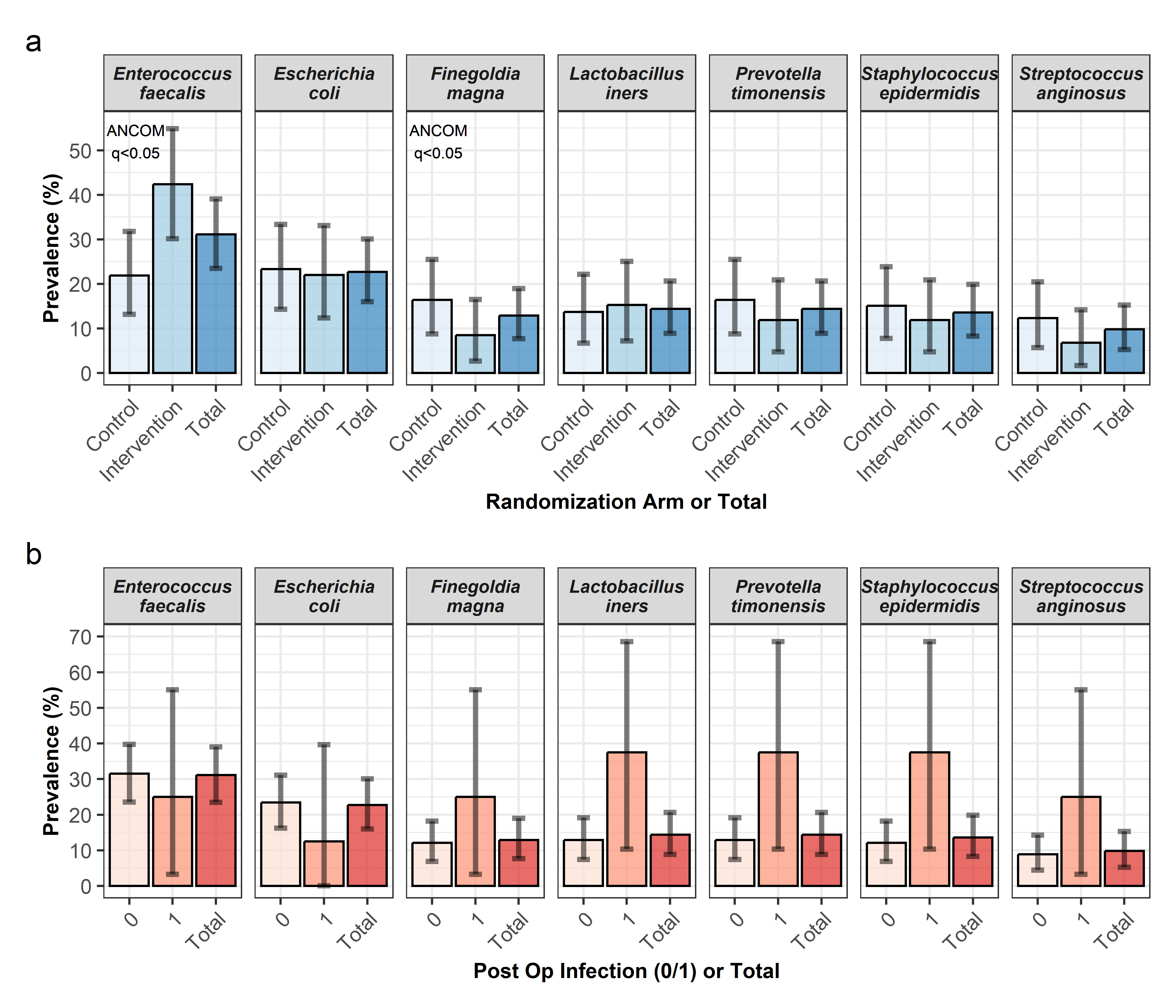


**Supplementary Figure 3.** Prevalence is shown for bacteria detected in at least 10% of NGS positive samples by 16S rRNA gene sequencing. Bacteria were screened by ANCOM for differential abundance between (A) randomization arms and (B) subsets developing post operative infection after 14 days. Binomial 95% confidence intervals were estimated using the R library ‘binom’.
